# Hydrolysis of Methionine- and Histidine-Containing Peptides Promoted by Dinuclear Platinum(II) Complexes with Benzodiazines as Bridging Ligands: Influence of Ligand Structure on the Catalytic Ability of Platinum(II) Complexes

**DOI:** 10.1155/2018/3294948

**Published:** 2018-05-08

**Authors:** Snežana Rajković, Beata Warżajtis, Marija D. Živković, Biljana Đ. Glišić, Urszula Rychlewska, Miloš I. Djuran

**Affiliations:** ^1^Department of Chemistry, Faculty of Science, University of Kragujevac, R. Domanovića 12, 34000 Kragujevac, Serbia; ^2^Faculty of Chemistry, Adam Mickiewicz University, Umultowska 89B, 61-614 Poznań, Poland; ^3^Department of Pharmacy, Faculty of Medical Sciences, University of Kragujevac, Svetozara Markovića 69, 34000 Kragujevac, Serbia; ^4^Serbian Academy of Sciences and Arts, Knez Mihailova 35, 11000 Belgrade, Serbia

## Abstract

Dinuclear platinum(II) complexes, [{Pt(en)Cl}_2_(*μ*-qx)]Cl_2_·2H_2_O (**1**), [{Pt(en)Cl}_2_(*μ*-qz)](ClO_4_)_2_ (**2**), and [{Pt(en)Cl}_2_(*μ*-phtz)]Cl_2_·4H_2_O (**3**), were synthesized and characterized by different spectroscopic techniques. The crystal structure of **1** was determined by single-crystal X-ray diffraction analysis, while the DFT M06-2X method was applied in order to optimize the structures of **1–3**. The chlorido Pt(II) complexes **1–3** were converted into the corresponding aqua species **1a–3a**, and their reactions with an equimolar amount of Ac–L–Met–Gly and Ac–L–His–Gly dipeptides were studied by ^1^H NMR spectroscopy in the pH range 2.0 < pH < 2.5 at 37°C. It was found that, in all investigated reactions with the Ac–L–Met–Gly dipeptide, the cleavage of the Met–Gly amide bond had occurred, but complexes **2a** and **3a** showed lower catalytic activity than **1a**. However, in the reactions with Ac–L–His–Gly dipeptide, the hydrolysis of the amide bond involving the carboxylic group of histidine was observed only with complex **1a**. The observed disparity in the catalytic activity of these complexes is thought to be due to different relative positioning of nitrogen atoms in the bridging qx, qz, and phtz ligands and consequent variation in the intramolecular separation of the two platinum(II) metal centers.

## 1. Introduction

Selective cleavage of peptides and proteins is an important procedure in biochemistry and molecular biology. However, the extreme kinetic inertness of the amide bond, with an estimated half-life between 250 and 600 years under physiological conditions [1], makes this procedure particularly interesting from the chemical point of view. This remarkable kinetic stability of the amide bond is required for its function but presents a challenge when there is a physiological need to break it. Typical proteolytic enzymes such as carboxypeptidase A contain a zinc(II) ion in their active sites, thus suggesting that small metal coordination complexes may play the role of a protease [2]. In recent years, a number of metal complexes including zinc(II) [3, 4], cobalt(III) [5], iron(II/III) [6–9], copper(II) [10–12], nickel(II) [13, 14], cerium(IV) [15], and zirconium(IV) [16, 17] have been found to be effective at promoting the hydrolysis of unactivated amide bonds in peptides and proteins. Besides the basic requirement that the complex should be able to promote amide bond cleavage, the selectivity of the cleavage remains a big challenge. In recent years, a great deal of interest has focused on the study of the interactions of mononuclear Pt(II) [18–20] and Pd(II) [19–33] complexes with methionine- and histidine-containing peptides. These interactions are of cardinal importance after the discovery that aqua derivatives of the investigated Pt(II) and Pd(II) complexes can be promising reagents for the hydrolytic cleavage of the abovementioned peptides. In general, it was shown that these complexes bind to the heteroatom in the side chain of methionine [18–25] or histidine [19, 20, 26–34] and promote cleavage of the amide bond involving the carboxylic group of the anchoring amino acid. Recent studies in one of our laboratories showed that dinuclear diazine-bridged Pt(II) complexes are very efficient in promoting the hydrolysis of the amide bond in methionine-containing peptides [35]. It was found that two Pt(II) ions bridged with one aromatic pyrazine (pz) ligand are more efficient in the hydrolysis of the methionine-containing peptides than a single Pt(II) ion in the corresponding mononuclear complex. The better catalytic activity of pyrazine-bridged dinuclear Pt(II) complexes in comparison with the corresponding mononuclear complexes has been explained by possible cooperation between two pyrazine-bridged Pt(II) ions. The cooperation between two metal ions as an important advantage of their dimerization was also demonstrated in the hydrolysis of DNA, RNA, and their models catalyzed by polynuclear metal complexes and metalloenzymes [36–43]. More recently, we have compared catalytic properties of two aqua dinuclear Pt(II) complexes with those of pyrazine and pyridazine (pydz) ligands, [{Pt(en)(H_2_O)}_2_(*μ*-pz)]^4+^ and [{Pt(en)(H_2_O)}_2_(*μ*-pydz)]^4+^, with peptides containing amino acids methionine and/or histidine in the side chain. The results obtained from that study showed that the [{Pt(en)(H_2_O)}_2_(*μ*-pydz)]^4+^ complex reacts only with methionine sulfur atom and promotes the sole cleavage of the amide bond involving the carboxylic group of this anchoring amino acid, while the analogous pyrazine Pt(II) aqua dimer reacts with both residues, promoting cleavage of the amide bonds involving the carboxylic groups of both amino acids, methionine and histidine [35, 44]. The X-ray data for the corresponding chloride complexes, [{Pt(en)Cl}_2_(*µ*-pydz)]Cl_2_ and [{Pt(en)Cl}_2_(*µ*-pz)]Cl_2_, confirmed the hidden position of the Pt(II) centers in the pydz-bridged Pt(II) complex caused by their close proximity due to the *ortho*-positioning of the two diazine nitrogen atoms (the two Pt(II) centers were only 3.2535(4) Å apart), compared to the separation of 6.7890(3) Å in the analogous pz-bridged Pt(II) dimer.

As a continuation of our interest in the reactions between methionine- and histidine-containing peptides and dinuclear Pt(II) complexes with six-membered aromatic diazines as bridging ligands, in the present study, we describe synthesis and characterization of three new dinuclear platinum(II) complexes, [{Pt(en)Cl}_2_(*μ*-qx)]Cl_2_·2H_2_O (**1**), [{Pt(en)Cl}_2_(*μ*-qz)](ClO_4_)_2_ (**2**), and [{Pt(en)Cl}_2_(*μ*-phtz)]Cl_2_·4H_2_O (**3**) (qx = quinoxaline, qz = quinazoline, phtz = phthalazine, and en = ethylenediamine). Complexes were characterized by NMR (^1^H and ^13^C), UV-Vis, and IR spectroscopic techniques. The crystal structure of **1** was determined by single-crystal X-ray diffraction analysis, while the DFT M06-2X method was applied in order to optimize the structures of **1–3**. The complexes **1–3** were converted into the corresponding aqua derivatives, and their catalytic activities in the hydrolysis of the *N*-acetylated Ac–L–His–Gly and Ac–L–Met–Gly dipeptides were investigated.

## 2. Materials and Methods

### 2.1. Materials

Distilled water was demineralized and purified to a resistance greater than 10 MΩ·cm. The compounds D_2_O, DNO_3_, NaOD, ethylenediamine (en), quinoxaline (1,4-benzodiazine, qx), quinazoline (1,3-benzodiazine, qz), phthalazine (2,3-benzodiazine, phtz), and K_2_[PtCl_4_] were obtained from Sigma-Aldrich Chemical Co. All common chemicals were of reagent grade. The dipeptides L-histidyl-glycine (L-His–Gly) and L-methionyl-glycine (L-Met–Gly) were obtained from Bachem A.G. The terminal amino group in these dipeptides was acetylated by a standard method [21]. The [Pt(en)Cl_2_] complex was synthesized according to a procedure published in the literature [28, 45, 46]. The purity of the complex was checked by elemental microanalyses and NMR (^1^H and ^13^C) spectroscopy.

### 2.2. Synthesis of Dinuclear Platinum(II) Complexes **1–3**

The complexes [{Pt(en)Cl}_2_(*μ*-qx)]Cl_2_·2H_2_O (**1**), [{Pt(en)Cl}_2_(*μ*-qz)](ClO_4_)_2_ (**2**), and [{Pt(en)Cl}_2_(*μ*-phtz)]Cl_2_·4H_2_O (**3**) were synthesized by modification of the procedure published in the literature [35, 44, 47, 48].

The mononuclear [Pt(en)Cl_2_] complex was converted into the corresponding monodimethylformamide complex [Pt(en)(dmf)Cl]NO_3_ by treatment with 0.98 equivalent of AgNO_3_. The suspension of 0.1487 g (0.456 mmol) of [Pt(en)Cl_2_] in 10.0 mL of dimethylformamide (dmf) was added to the solution of 0.0759 g (0.447 mmol) of AgNO_3_ in 5 mL of dmf. The mixture was stirred overnight at room temperature in the dark. The precipitated AgCl was removed by filtration, and resulting pale yellow dmf solution of [Pt(en)(dmf)Cl]NO_3_ was used as the starting material for the preparation of the required quinoxaline-, quinazoline-, or phthalazine-bridged platinum(II) complexes.

The dmf solution of the ligand L (0.0297 g; 0.228 mmol) (L is quinoxaline, qx; quinazoline, qz; and phthalazine, phtz) was added dropwise to the solution of [Pt(en)(dmf)Cl]NO_3_ complex. The mixture was stirred at room temperature in the dark for 24 h. The solvent was then rotary evaporated, and the residue was washed with ether. The crude product was dissolved in a minimal amount of aqueous solution of LiCl (0.5 M) for **1** and **3** or LiClO_4_ (0.5 M) for **2**. The obtained solutions were left overnight in the dark. The pale yellow precipitate of the dinuclear platinum(II) complex was removed by filtration, washed with methanol and then ether, and air-dried. Depending on the type of the bridging ligand L, the yield of complexes **1–3** was between 35 and 40%. Elemental analysis for **1** (Pt_2_C_12_H_26_N_6_Cl_4_O_2_): found: C, 17.87; H, 3.20; and N, 10.15%; calc.: C, 17.61; H, 3.20; and N, 10.27%. ^1^H NMR (200 MHz, D_2_O): *δ* = 2.76 (m, 8H, en), 8.41 (dd, 2H, C6H and C7H, qx), 9.59 (s, 2H, C2H and C3H, qx), and 9.73 (dd, 2H, C5H and C8H, qx) ppm. ^13^C NMR (50 MHz, D_2_O): *δ* = 49.42 and 49.77 (en), 130.09 (C6, C7), 135.93 (C5, C8), 145.26 (C4a, C8a), and 151.77 (C2, C3) ppm. IR (KBr, ν, cm^−1^): ∼3415 (O-H stretch); 3275-3027 (N-H stretch); and 1631, 1597, 1587, and 1500 (C=N/C=C, quinoxaline group stretch). UV-Vis (H_2_O, *λ*_max_, nm): 247 (*ε* = 2.5·10^4^ M^−1^·cm^−1^) and 339 (*ε* = 9.8·10^3^ M^−1^·cm^−1^). Elemental analysis for **2** (Pt_2_C_12_H_22_N_6_Cl_4_O_8_): found: C, 15.63; H, 2.60; and N, 8.94%; calc.: C, 15.83; H, 2.44; and N, 9.23%. ^1^H NMR (200 MHz, D_2_O): *δ* = 2.81 (m, 8H, en), 8.16 (m, 2H, C6H and C7H, qz), 8.51 (m, 2H, C5H and C8H, qz), 9.52 (d, H, C4H, qz), and 10.12 (d, H, C2H, qz) ppm. ^13^C NMR (50 MHz, D_2_O): *δ* = 50.24 (en), 128.58 (C4a), 130.14 (C5), 132.81 (C8), 135.11 (C6), 143.10 (C7), 152.39 (C8a), 168.56 (C4), and 178.11 (C2) ppm. IR (KBr, ν, cm^−1^): 3233 and 3138 (N-H stretch); 1620 and 1587 (C=N/C=C quinazoline group stretch); and 1096, 1062, and 623 (perchlorate counterion). UV-Vis (H_2_O, *λ*_max_, nm): 237 (*ε* = 4.7·10^4^ M^−1^·cm^−1^) and 328 (*ε* = 8.1·10^3^ M^−1^·cm^−1^). Elemental analysis for **3** (Pt_2_C_12_H_30_N_6_Cl_4_O_4_): found: C, 16.45; H, 3.58; and N, 9.57%; calc.: C, 16.87; H, 3.54; and N, 9.84%. ^1^H NMR (200 MHz, D_2_O): *δ* = 2.83 (m, 8H, en), 8.40 (m, 4H, C5H, C6H, C7H, and C8H, phtz), and 10.05 (s, 2H, C1H and C4H, phtz) ppm. ^13^C NMR (50 MHz, D_2_O): *δ* = 51.27 (en), 130.96 (C4a, C8a), 131.04 (C5, C8), 140.50 (C6, C7), and 164.32 (C1, C4) ppm. IR (KBr, ν, cm^−1^): ∼3489 (O-H stretch); 3156 and 3046 (N-H stretch); and 1625 (C=N/C=C, phthalazine group stretch). UV-Vis (H_2_O, *λ*_max_, nm): 236 (*ε* = 5.1·10^4^ M^−1^·cm^−1^) and 329 (*ε* = 8.7·10^3^ M^−1^·cm^−1^).

### 2.3. Preparation of Aqua Platinum(II) Complexes **1a–3a**

The dinuclear chlorido complexes **1–3** were converted into the corresponding aqua derivatives, [{Pt(en)(H_2_O)}_2_(*μ*-L)]^4+^, where L = qx (**1a**), qz (**2a**), and phtz (**3a**), according to a previously published method [49] by treatment with 3.98 (complexes **1** and **3**) and 1.98 (complex **2**) equivalents of AgNO_3_. In each case, the precipitated white solid product was removed by filtration in the dark, and the fresh solutions of the aqua complexes **1a–3a** were kept in a refrigerator and used in the further experiments.

### 2.4. Measurements

Elemental microanalyses for carbon, hydrogen, and nitrogen parameters were performed by the Microanalytical Laboratory, Faculty of Chemistry, University of Belgrade. All pH measurements were realized at ambient temperature using a Mettler Toledo SevenCompact S220-U pH meter calibrated with Mettler Toledo-certified buffer solutions of pH 4.00 and 7.00. The results were not corrected for the deuterium isotope effect. The UV-Vis spectra were recorded on a Shimadzu double-beam spectrophotometer equipped with thermostated 1.00 cm quartz Suprasil cells after dissolving the corresponding platinum(II) complex in water over the wavelength range of 200–500 nm. The concentration of the dinuclear platinum(II) complexes was 5 × 10^−5^ M. The IR spectra were recorded on a PerkinElmer Spectrum One FT-IR spectrometer using the KBr pellet technique, over the range of 4000–450 cm^−1^. The NMR spectra of platinum(II) complexes **1–3** and aromatic *N*-heterocyclic ligands were recorded at 25°C in D_2_O-containing TSP (sodium 3-(trimethylsilyl)propionate) as the internal reference on a Varian Gemini 2000 spectrometer (^1^H at 200 MHz; ^13^C at 50 MHz). Chemical shifts are reported in parts per million (ppm), and scalar couplings are reported in hertz (Hz). 0.005 g of each compound was dissolved in 0.6 mL of D_2_O, and this solution was transferred into a 5 mm NMR tube. All the NMR spectra were processed using the Varian VNMR software (version 6.1, revision C). Fresh solutions of the aqua derivatives of dinuclear platinum(II) complexes **1a–3a** and Ac–L–Met–Gly and Ac–L–His–Gly dipeptides were prepared separately and then mixed in 1 : 1 molar ratio. The initial concentrations of dipeptide and aqua complex solutions were 20 mM. All reactions were performed in the pH range 2.0 < pH < 2.5 at 37 °C.

### 2.5. Crystallographic Data Collection and Refinement of the Structure of **1**

Diffraction data for [{Pt(en)Cl}_2_(*μ*-qx)]Cl_2_·2H_2_O complex (**1**) were measured with an Xcalibur kappa geometry diffractometer using CrysAlisPro software [50] and monochromated Mo K*α* radiation (*λ* = 0.71073 Å). Crystal data and experimental details are summarized in Table S1. The structure was solved by direct methods using SHELXS-86 [51] and refined by full-matrix least-squares calculations on *F* ^2^ with SHELXL [52]. The intensity data were corrected for absorption effects [50]. Anisotropic displacement parameters were refined for all nonhydrogen atomic positions. Hydrogen atoms attached to the carbon and nitrogen atoms were placed in calculated positions (methylene C–H = 0.97, aromatic C–H = 0.93, and amine N–H = 0.89 Å). Water hydrogens have been located on subsequent difference Fourier maps, and their bond lengths were standardized to a value of 0.85 Å. During the refinement, isotropic displacement parameters for H atoms were assigned to be 20% higher than the isotropic equivalent of the atom to which the H atom was bonded. All H atoms were refined as riding. MERCURY [53] was used to prepare drawings. Selected bond distances and angles are reported in Table S2. Geometrical parameters describing intermolecular hydrogen bonds and stacking interactions are listed in Tables S3 and S4, respectively.

### 2.6. Quantum-Mechanical Methods

The M06-2X functional [54] in combination with the cc-pVTZ basis set [55, 56] for the H, C, N, and Cl atoms and the LanL2TZ(f) basis set [57] for the platinum atoms was used to optimize the geometries of the studied systems. M06-2X is a hybrid metadensity functional and has been recommended for the main group and transition metals thermochemistry and kinetics [58]. To take into account the effect of the solvent, the polarizable continuum model (PCM) [59] was used with water chosen as the solvent. All structures were fully optimized without any geometric constraints. The optimized structures were confirmed to be potential energy minima by vibrational frequency calculations at the same level of theory because no imaginary frequencies were found.

All DFT calculations were performed using the Gaussian 09 program package [60]. The M06-2X method and cc-PVTZ basis set were employed as implemented in the software package, while the LanL2TZ(f) basis set for platinum was obtained from EMSL Basis Set Exchange (https://bse.pnl.gov/bse/portal).

## 3. Results and Discussion

### 3.1. Synthesis and Structural Features of the Dinuclear Platinum(II) Complexes **1–3**

**Figure 1:**
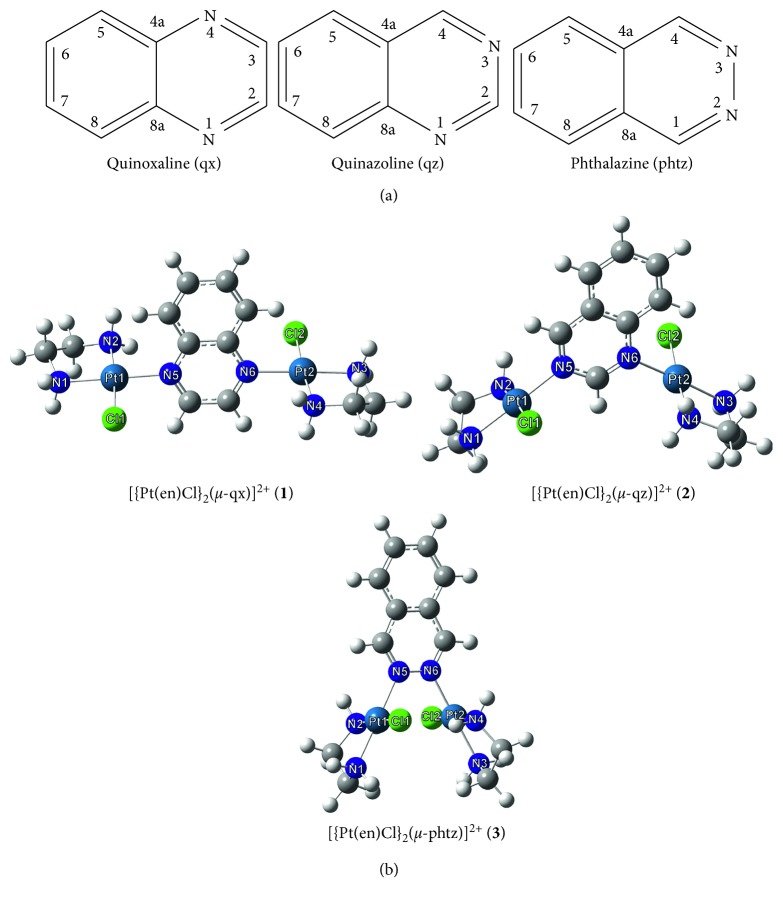
Schematic drawing of the *N*-heterocycles used in this study (a) and the structures of dinuclear platinum(II) complexes **1–3** calculated at the M06-2X(PCM)/cc-pVTZ+LanL2TZ(f) level of theory. The numbering scheme of carbon atoms in *N*-heterocycles is in agreement with IUPAC recommendations for the fused ring system and does not match the one applied in the X-ray study of **1**.

Three aromatic *N*-heterocycles, quinoxaline (qx), quinazoline (qz), and phthalazine (phtz) (Figure 1(a)), were used as the bridging ligands between two {Pt(en)Cl} units. All these *N*-heterocycles contain two nitrogen atoms within one ring but at different positions, that is, 1,4 for qx, 1,3 for qz, and 2,3 for phtz, resulting in their different steric and electronic properties. However, despite these differences, they all reacted with Pt(II) ion to form dinuclear species [{Pt(en)Cl}_2_(*μ*-qx)]Cl_2_·2H_2_O (**1**), [{Pt(en)Cl}_2_(*μ*-qz)](ClO_4_)_2_ (**2**), and [{Pt(en)Cl}_2_(*μ*-phtz)]Cl_2_·4H_2_O (**3**) (en is a bidentate-coordinated ethylenediamine). The stoichiometries of **1–3** were confirmed by elemental microanalysis, and the structures emerged from NMR (^1^H and ^13^C), IR, and UV-Vis spectroscopic methods. Crystals of **1** were obtained after the crude product, resulting from the reaction of mononuclear [Pt(en)Cl_2_] and quinoxaline, was dissolved in a minimal amount of water saturated with LiCl; the crystal structure of this complex was determined by single-crystal X-ray analysis. Simultaneous attempts to crystallize complexes **2** and **3** from their amorphous powders using different solvents (water, methanol, acetone, chloroform, and dimethylformamide) were unsuccessful. Accordingly, the structures of the complexes **1–3** have been optimized by means of the DFT M06-2X method.

#### 3.1.1. NMR Characterization

Ambient temperature NMR spectra of platinum(II) complexes **1–3** and the corresponding *N*-heterocyclic ligands were measured in D_2_O. The ^1^H and ^13^C NMR chemical shifts as well as Δ(^1^H)_coord_ and Δ(^13^C)_coord_ coordination shifts for **1–3** determined in respect to those for the uncoordinated *N*-heterocycles are listed in Table 1.

The chemical shifts of the used *N*-heterocycles in D_2_O are almost identical with those reported in literature for the NMR spectra of these compounds measured in other solvents [61, 62]. In the aromatic region, the ^1^H NMR spectra of **1** and **2** consist of two multiplets corresponding to the protons of the condensed benzene ring (C5H and C8H, and C6H and C7H, resp.), with the chemical shifts significantly differing from those of the uncoordinated qx and qz ligands. Contrary to this, all benzene protons C5H–C8H in phtz-containing complex **3** give rise to one multiplet. Besides the resonances corresponding to the benzene ring, those due to the protons of the diazine ring can be detectable in the aromatic region of the ^1^H NMR spectra. Thus, a singlet due to the C2H and C3H, and C1H and C4H for **1** and **3**, respectively, is observed in their spectra. On the other hand, diazine protons C2H and C4H for qz-containing complex **2** are nonequivalent, which results in the appearance of the two doublets. In addition to the characteristic ^1^H NMR resonances for the aromatic protons of the complexes **1–3**, the aliphatic CH_2_ group of the bidentatedly coordinated en ligand gives a singlet in the region 2.78–2.84 ppm. This singlet is on the same chemical shift as that for the mononuclear [Pt(en)Cl_2_] complex.

As can be seen from Table 1, the resonances for the aromatic protons of the complexes **1–3** are downfield shifted with respect to those for the uncoordinated *N*-heterocycles. The downfield shifting for the protons in the used *N*-heterocycles after their platination can be ascribed to a delocalization of the charge deficiency (cation formation by Pt(II) coordination) throughout all the rings in the molecules as anticipated [61, 63]. It is important to note that the ^1^H chemical shifts for the complexes **2** and **3** which contain quinazoline- and phthalazine-bridging ligands, respectively, are in agreement with those for the structurally similar platinum(II) complexes reported previously [64, 65].

The ^13^C NMR spectra of **1–3** in D_2_O due to the aromatic carbons display four (**1** and **3**) and eight (**2**) distinct signals and are noticeably different from those of the free *N*-heterocyclic ligands; their addition to the D_2_O solution of the complexes results in the appearance of another set of ^13^C signals (Table 1). As a consequence of the Pt(II) complexation of the investigated *N*-heterocyclic ligands, all ring carbons are deshielded (up to +14.72 ppm for C2 in complex **2**). The chemical shift of the methylene carbons of ethylenediamine in **1–3** is identical to that of these carbons of [Pt(en)Cl_2_] complex (*δ* = 49.42–51.27 ppm).

#### 3.1.2. IR and UV-Vis Characterization

The IR and UV-Vis spectral data for the dinuclear platinum(II) complexes **1–3** are listed in the Materials and Methods (*vide infra*). The IR spectra of the complexes measured in the wavenumber range of 4000–450 cm^−1^ show the bands attributable to the vibration of the coordinated *N*-heterocyclic ligand, as well as those due to the bidentatedly coordinated en ligand, crystalline water molecules (**1** and **3**), and perchlorate counteranion (**2**). Thus, a broad absorption at ∼3400 cm^−1^ is assigned to the stretching vibration of the OH group and confirms the presence of crystalline water molecules in **1** and **3** [66]. Besides, the complexes **1–3** exhibit two very strong and sharp bands at ∼3200 and 3100 cm^−1^, which were assigned, respectively, to the asymmetric and symmetric stretching vibrations of the coordinated amino group of en ligand [67]. Complex **2** exhibits a very strong band with two submaxima at 1096 and 1062 cm^−1^ and a strong one at 623 cm^−1^ which can be attributed to the ν(ClO) and *δ*(OClO) modes, respectively, of the uncoordinated perchlorates [68]. The presence of the two bands attributed to the asymmetric stretching vibration of ClO_4_^−^ ion can be the consequence of its participation in hydrogen bonding interactions, which results in the lowering of the point group symmetry from *T*_*d*_ to *C*_2*ν*_, leading to “pseudomonodentate” spectroscopic behaviour of ClO_4_^−^ [68, 69].

The shape of UV-Vis spectra and the values of *λ*_max_ are similar for **1–3**, indicating the same bidentate-bridging coordination mode of the corresponding *N*-heterocycle to the Pt(II) ion (Figure S1 in the Supplementary Material). In all complexes, the absorbance peaks at higher energy are due to *π*→*π* ^∗^ transitions in the aromatic *N*-heterocycles [70], and they show significant red shifts compared to those in the free ligands. The second absorbance peak at ∼330 nm observed for the complexes corresponds to LMCT (ligand-to-metal charge transfer) transition.

#### 3.1.3. Description of the Crystal Structure of **1**

**Figure 2:**
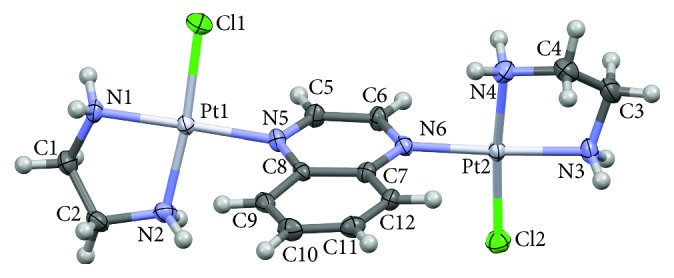
Perspective view of the dinuclear complex cation [{Pt(en)Cl}_2_(*μ*-qx)]^2+^ in the crystal of **1** at 295 K. Atomic displacement ellipsoids are drawn at the 40% probability level.

The molecular structure and labeling scheme of **1** are shown in Figure 2. X-ray analysis has confirmed that the [{Pt(en)Cl}_2_(*μ*-qx)]Cl_2_·2H_2_O complex is a dinuclear complex of Pt(II) bridged by the quinoxaline ligand. Each Pt(II) ion exhibits an approximately square planar coordination, with one Pt–Cl bond, one Pt–N bond to the qx ligand, and two Pt–N bonds to the same chelating diamine (en) ligand. The Pt⋯Pt distance is 6.8217(7) Å, comparable with the mean value of 6.82(6) Å obtained from 47 observations (24 hits) for crystal structures containing pyrazine-bridged Pt(II) fragments, deposited in the CSD [71]. Two Cl ligands and two en chelate rings are mutually *trans* oriented. The two Pt–N(en) distances within each en chelate slightly differ; the bond that is *trans* to the Pt–Cl bond is consistently longer than the one that is *trans* to the Pt–N(qx) bond (average values 2.035(1) Å versus 2.013(3) Å). The coordinated diazine ring of qx is significantly inclined with respect to the Pt(II) coordination planes. The dihedral angles between the heterocyclic ring plane and the square plane around each of the two Pt(II) ions amount to 72.76(16)° and 89.79(16)°. Noticeably, in the centrosymmetric [{Pt(en)Cl}_2_(*μ*-pz)]Cl_2_ complex, the dihedral angle between the pyrazine ring plane and the square plane around Pt(II) amounts to only 58.4(1) Å [35]. The diamine rings adopt the usual twist conformation of the same helicity within one complex molecule. The quinoxaline part is not strictly planar; the two rings are inclined at 4.0(2)°. There are relatively short intramolecular Pt⋯H–C_benzene_ contacts of 2.81 and 2.75 Å to Pt1 and Pt2 ions, respectively, which might be connected with a noticeable twisting of the benzene moiety with respect to the Pt–N⋯N–Pt line. The quoted H⋯Pt distances belong to the shortest within the platinum(II) complexes with pyrazine and related ligands [71].

**Figure 3:**
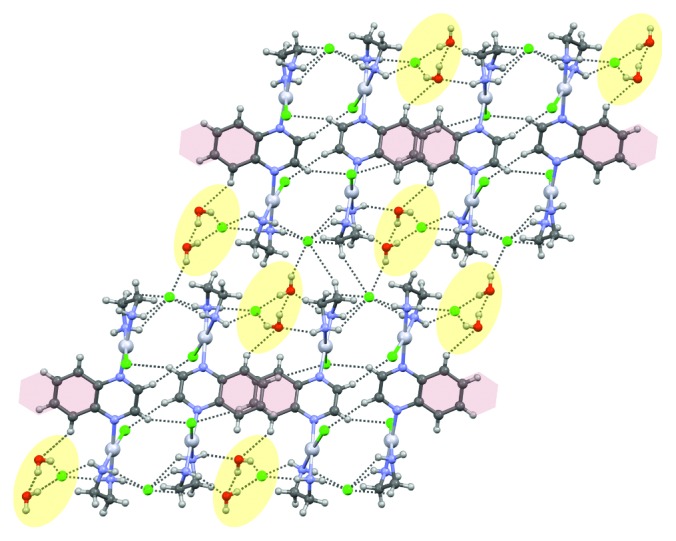
Arrangement of molecules constituting the multicomponent crystal of [{Pt(en)Cl}_2_(*μ*-qx)]Cl_2_·2H_2_O (**1**). Hydrogen bonds are marked by dotted lines, and *π*⋯*π* interactions operate between partially overlapping molecules that stack along the *b*-direction. Structural voids filled by water molecules and chloride ions are shaded in yellow, while benzene rings involved in stacking interactions are shaded in purple.

The crystal packing of **1** is driven by numerous hydrogen bonds of the NH···Cl, NH···O, OH···Cl, and OH···O type (for geometrical parameters describing these interactions, see Table S3 in the Supplementary Material), supplemented by CH···Cl and CH···O intermolecular interactions. The complex cations form double-molecular columns, extending along the *b*-direction, within which the aromatic rings are involved in weak *π*···*π* interactions (geometrical parameters describing these interactions are included in Table S4). The neighbouring Pt(II) ions along the *b* direction are at a distance of 6.4872(3) Å, while the shortest intermolecular Pt⋯Pt distance is only 4.6100(5) Å (Pt1⋯Pt2 at 0.5 − *x*, 0.5 + y, and 0.5 − z) and operates between molecules that are doubly bridged by an uncoordinated Cl3 ion acting as an acceptor of two relatively strong N-H···Cl hydrogen bonds (Table S3; Figure 3). The uncoordinated chloride anions and water molecules are located in crystal voids (Figure 3). The presence of water molecules in this crystal structure can be contrasted with the absence of any solvent molecules in the crystal structures of related Pt(II) complexes, namely, [{Pt(en)Cl}_2_(*μ*-pz)]Cl_2_ [35] and [{Pt(en)Cl}_2_(*μ*-pydz)]Cl_2_ [44]. Clearly, the incorporation of an extra benzene ring to the ligand causes noticeable difficulties in close packing of the complex cationic species and consequent inclusion of water molecules in order to fill structural voids. It also causes replacement of anion···*π* interactions present in the crystal structure of [{Pt(en)Cl}_2_(*μ*-pz)]Cl_2_ by parallel-displaced slightly attractive *π*···*π* stacking interactions (Figure 3).

#### 3.1.4. Computational Studies

The structures of the dinuclear platinum(II) complexes **1–3** were optimized in water at the M06-2X(PCM)/cc-pVTZ + LanL2TZ(f) level of theory. The optimized structures of **1–3** are shown in Figure 1(b), and the values of the calculated bond lengths and angles are presented in Table S2. This table also compares the DFT-calculated parameters for complex **1** to those inferred from the corresponding X-ray structure. As can be seen, the calculated bond lengths and angles for this complex show very good agreement with the corresponding X-ray data. This supports our recently obtained results showing that the M06-2X method can reproduce to a reasonable good extent the experimental structural parameters for platinum(II) complexes with aromatic *N*-heterocycles [72]. In addition, this method has been shown as appropriate for evaluation of the electron-donating properties of aromatic *N*-heterocycles [73]. The error of the calculated Pt–N and Pt–Cl bond lengths was found to be ∼0.03 and 0.04 Å, respectively, whereas calculations reproduced very well nonequivalency of the two Pt–N(en) bonds, that is, the Pt–N(en) bond which is in the *trans* position to the coordinated nitrogen atom of qx and the Pt–N(en) bond which is slightly shorter than the remaining Pt–N(en) bonds (Table S2). Moreover, the calculated N1–Pt1–N2 and N3–Pt2–N4 angles in the five-membered chelate rings show good agreement with the experimentally found ones; these angles (approximately 83°) significantly deviate from the ideal angle of 90°.

From the DFT study, we found that the complexes **2** and **3** have the same square planar geometry as crystallographically characterized complex **1** (Figure 1(b)). These complexes contain two Pt(II) ions, which are coordinated to two nitrogen atoms of the chelating en ligand, nitrogen of the bridging qz and phtz ligands, and chlorine in the fourth coordination site. The Pt–N and Pt–Cl bond lengths in these complexes are in the expected range and compare well with those in **1** and the other platinum(II) complexes with the same [N_3_Cl] coordination sphere, such as [{Pt(en)Cl}_2_(*μ*-pz)]^2+^ and [{Pt(en)Cl}_2_(*μ*-pydz)]^2+^ [35, 44]. Furthermore, the calculations for **2** and **3** provide the same difference between the Pt–N(en) bond lengths as in **1**, as well as significant deviation of the N1–Pt1–N2 and N3–Pt2–N4 angles within the five-membered chelate rings from the angle of 90°.

Similarly to [{Pt(en)Cl}_2_(*μ*-pydz)]^2+^ complex, in which the intramolecular distance between two Pt(II) ions was found to be 3.2535(4) Å [44], in [{Pt(en)Cl}_2_(*μ*-phtz)]^2+^ (**3**), two Pt(II) ions are only 3.2392 Å apart. It is important to note that the aromatic *N*-heterocycles, pyridazine and phthalazine, which act as bridging ligands in these two complexes, contain two nitrogen atoms in the *ortho*-position. The intramolecular distance between two Pt(II) ions in **3** is shorter than the sum of van der Waals radii for two Pt(II) ions which amounts to 3.50 Å [74]. On the other hand, this distance in **1** and **2** is calculated to be 6.8596 and 5.7914 Å, respectively, and is significantly longer than the sum of van der Waals radii for Pt(II) ions. This is a direct consequence of the *para*- and *meta*-positions of the two nitrogen donors in qx and qz ligands, respectively.

### 3.2. Reactions of Aqua Derivatives of Dinuclear Platinum(II) Complexes **1–3** with Ac–L–Met–Gly and Ac–L–His–Gly Dipeptides

**Figure 4:**
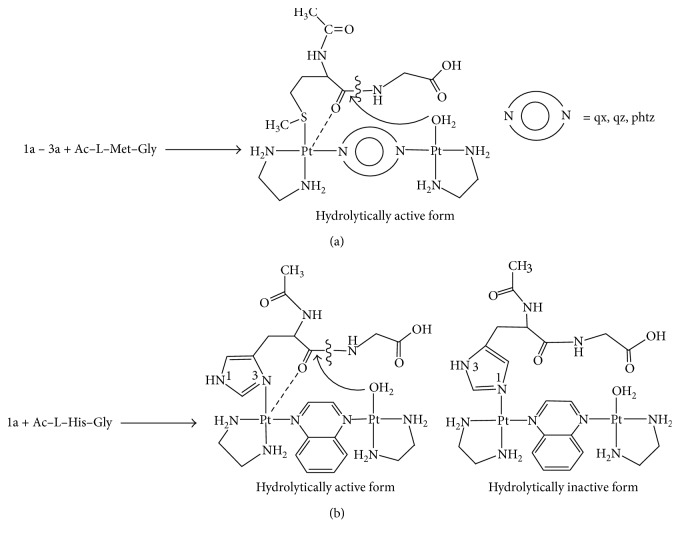
Schematic presentation of the hydrolytic reaction of Ac–L–Met–Gly (a) and Ac–L–His–Gly (b) in the presence of complexes **1a–3a** in the pH range 2.0 < pH < 2.5 in D_2_O as a solvent and at 37°C. In the reaction with Ac–L–His–Gly, only 1a was catalytically active complex.

The chlorido Pt(II) complexes **1–3** were converted into the corresponding aqua species **1a–3a**, [{Pt(en)(H_2_O)}_2_(*μ*-qx)]^4+^ (**1a**), [{Pt(en)(H_2_O)}_2_(*μ*-qz)]^4+^ (**2a**), and [{Pt(en)(H_2_O)}_2_(*μ*-phtz)]^4+^ (**3a**), and their reactions with Ac–L–Met–Gly and Ac–L–His–Gly were studied by ^1^H NMR spectroscopy. Complexes **1a–3a** and the corresponding dipeptide were reacted in 1 : 1 molar ratio, and all reactions were performed in the pH range 2.0 < pH < 2.5 at 37°C. In the reaction between **1a–3a** and Ac–L–Met–Gly, only one Pt(II)–peptide product was obtained. In this product, complexes **1a–3a** are monodentatedly coordinated to the dipeptide through its methionine sulfur atom (Figure 4(a)). The binding of **1a–3a** to the methionine side chain was observed by the simultaneous decline of the resonance at 2.11 ppm due to the S-methyl protons of the free dipeptide and the growth of a resonance at 2.35–2.55 ppm, corresponding to the S-methyl protons of the dipeptide coordinated to platinum(II) [18–20]. The platinum(II)-dipeptide products formed in these reactions are intermediate species, and they promote hydrolysis of the Met–Gly amide bond in Ac–L–Met–Gly dipeptide.

**Figure 5:**
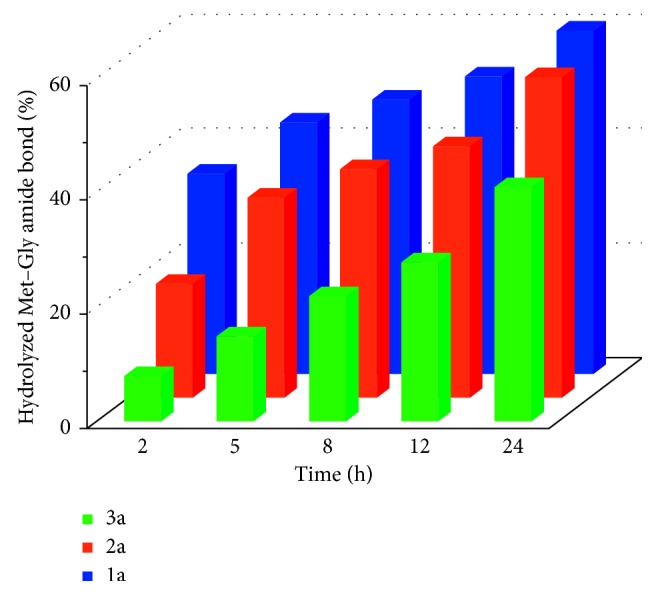
Time dependence of the hydrolytic cleavage of the Met–Gly amide bond in Ac–L–Met–Gly dipeptide in the presence of equimolar amount of **1a**–**3a** complexes in the pH range 2.0 < pH < 2.5 and at 37°C.

The amount of the hydrolyzed Met–Gly amide bond in these reactions was determined by integration of the resonance for the glycine protons of the dipeptide attached to platinum(II) (4.02 ppm) and that for these protons of the free glycine (3.76 ppm). The changes in concentrations of the free glycine and nonhydrolyzed Ac–L–Met–Gly bound to platinum(II) were determined every 30 min during 24 h. During this time, the total amount of the platinum(II)-peptide product and free glycine was always equal to the initial concentration of Ac–L–Met–Gly dipeptide. The time dependence of the hydrolytic cleavage of the Met–Gly amide bond in the reaction between **1a–3a** and Ac–L–Met–Gly dipeptide is given in Figure 5. From this Figure, it can be concluded that the rate of the hydrolysis of the Ac–L–Met–Gly dipeptide resulting from the reaction with complexes **1a–3a** decreases in the following order: **1a** > **2a** > **3a**.

When an equimolar amount of complexes **1a–3a** was reacted with Ac–L–His–Gly dipeptide, under the abovementioned experimental conditions, no reaction was observed by proton NMR spectroscopy for the complexes **2a** and **3a** during 48 h. However, in the reaction between **1a** and this dipeptide, two platinum(II)-dipeptide products were observed in the reaction mixture after 30 min of reaction time (Figure 4(b)). These products were distinguished by observing the changes in the chemical shifts of two imidazole C2H and C5H protons with respect to those of free Ac–L–His–Gly dipeptide (*δ*_C2H_ = 8.61 and *δ*_C5H_ = 7.33 ppm). Additionally, the chemical shifts of these two protons for the investigated products were compared with those previously reported for the platinum(II)-peptide complexes obtained from the reactions of Ac–L–His–Gly with various dinuclear platinum(II) complexes [44, 75]. Based on this, we have found that the two platinum(II)-dipeptide products were constitutional isomers with a monodentate coordination of **1a** *via* either N3 (*δ*_C2H_ = 8.12 ppm and *δ*_C5H_ = 7.21 ppm) or N1 (*δ*_C2H_ = 7.95 ppm and *δ*_C5H_ = 6.89 ppm) atom to the imidazole ring. When the reaction between **1a** and Ac–L–His–Gly was monitored with time, a new signal at 3.64 ppm appeared in the ^1^H NMR spectrum which showed a tendency to increase during the passage of time. We have found that this signal belonged to the methylene protons of the free amino acid glycine. Upon addition of glycine to the reaction mixture, this resonance was enhanced. Additionally, the resonance at 3.83 ppm due to glycine protons of Ac–L–His–Gly dipeptide was decreasing gradually with the passing time. We assumed that these changes in the ^1^H NMR spectrum resulted from the hydrolysis of the His–Gly amide bond in Ac–L–His–Gly dipeptide. This assumption was based on our previous findings that only monodentate coordination of the dinuclear platinum(II) complex to the N3 imidazole nitrogen atom of histidine-containing peptides promotes cleavage of the amide bond involving the carboxylic group of the anchoring amino acid histidine [44, 75]. The concentrations of Ac–L–His–Gly and the hydrolysis products were determined from the known initial concentration of the dipeptide and from the integrated resonance of the free glycine. The cleavage of Ac–L–His–Gly was regioselective, and about 35% of the His–Gly bond in this dipeptide has cleaved after 48 h.

Complexes **1a–3a** showed different catalytic activities in the hydrolytic cleavage of Ac–L–Met–Gly and Ac–L–His–Gly dipeptides which resulted from the presence of different *N*-heterocycles as the bridging ligands in these complexes. In the presence of Ac–L–Met–Gly dipeptide, all complexes reacted with the methionine sulfur atom and cleaved the Met–Gly amide bond in this dipeptide, although with a different reaction rate. In a given time interval, the percentage of the hydrolyzed dipeptide varied depending on the type of the complex used for the reaction and decreased in the following order: **1a** > **2a** > **3a**. We compared catalytic activities of **1a** with [{Pt(en)(H_2_O)}_2_(*μ*-pz)]^4+^ [35] and **3a** with [{Pt(en)(H_2_O)}_2_(*μ*-pydz)]^4+^ [44] in the hydrolysis of Ac–L–Met–Gly dipeptide (Table 2). All reactions were performed under the same experimental conditions, in the pH range 2.0–2.5 and at 37°C. Complexes **1a** and [{Pt(en)(H_2_O)}_2_(*μ*-pz)]^4+^, with quinoxaline- and pyrazine-bridging ligands, respectively, have *para*-positioned nitrogen atoms. By contrast, complexes **3a** and [{Pt(en)(H_2_O)}_2_(*μ*-pydz)]^4+^, with phthalazine- and pyridazine-bridging ligands, respectively, have nitrogen atoms in an *ortho*-position. As can be seen from Table 2, complexes **1a** and **3a** showed lower catalytic activity than [{Pt(en)(H_2_O)}_2_(*μ*-pz)]^4+^ and [{Pt(en)(H_2_O)}_2_(*μ*-pydz)]^4+^ complexes, respectively. This can be attributed to the hindering role of the additional benzene ring in quinoxaline- and phthalazine-bridging ligands.

In the presence of Ac–L–His–Gly dipeptide, only complex **1a** reacted with the histidine side chain, promoting cleavage of the His–Gly amide bond, while the other two complexes **2a** and **3a** remained inactive. Obviously, this had to be connected with different positioning of the nitrogen atoms in the bridging qx, qz, and phtz ligands and consequent variation in intramolecular distances between the two platinum(II) ions in these dimetallic complexes. As mentioned above, the DFT calculated values for the intramolecular distances between two platinum(II) ions in the corresponding chlorido complexes **1–3** were 6.8596, 5.7914, and 3.2392 Å, respectively. One would expect these values to be nearly the same in complexes **1a–3a**, which are formed upon displacement of a chlorido ligand by a water molecule. The significantly shorter Pt⋯Pt distances in complexes **2a** and **3a** in comparison with **1a** indicate an increased steric crowding in the former two complexes, which is a direct cause of a total inhibition of the reaction with Ac–L–His–Gly and slower cleavage of the amide bond in Ac–L–Met–Gly dipeptides. Similarly to **1a**, the [{Pt(en)(H_2_O)}_2_(*μ*-pz)]^4+^ complex cation, with the pz-bridging ligand having two nitrogen atoms in *para*-position, reacts with the histidine side chain and promotes the cleavage of the His–Gly amide bond [35]. Like for **2a** and **3a**, no reaction with the histidine side chain was observed for [{Pt(en)(H_2_O)}_2_(*μ*-pydz)]^4+^ complex, with nitrogen atoms in the bridging ligands positioned in *ortho*-position of the aromatic ring [44].

## 4. Conclusions

Three isomeric six-membered aromatic diazines with an additional fused benzene ring, quinoxaline, quinazoline, and phthalazine, were shown as good bridging ligands between two {Pt(en)Cl} units forming dinuclear complexes **1–3**. From the DFT study, it was found that complexes **2** and **3** have the same square planar geometry as crystallographically characterized complex **1**. Compared to the [{Pt(en)Cl}_2_(*μ*-pz)]Cl_2_ parent compound [35], complex **1** contains an additional benzene ring and its hindering role is noticeable in the crystal structure on both molecular and supramolecular levels. The relative position of the two nitrogen atoms in the bridging aromatic diazine ring has a substantial effect on the catalytic activity of the aqua derivatives of the corresponding complexes **1–3** with methionine- and histidine-containing dipeptides. All complexes bind to the methionine side chain of Ac–L–Met–Gly and promote cleavage of the amide bond involving the methionine carboxylic group. However, only aqua platinum(II) complex with the quinoxaline-bridging ligand shows the catalytic ability in the reaction with Ac–L–His–Gly dipeptide. Our present results concerning dinuclear platinum(II) complexes with benzene-fused aromatic diazines as bridging ligands along with those previously reported for analogous platinum(II) complexes with pyrazine and pyridazine [35, 44, 48, 75] showed that selective cleavage of a peptide molecule containing both methionine and histidine side chains can be achieved by dinuclear platinum(II) complexes containing *ortho*- and *meta*-positioned nitrogen atoms in the aromatic diazine-bridging ligand.

## Figures and Tables

**Table 1:** NMR (^1^H and ^13^C) chemical and coordination shifts (*δ*, ppm), alongside multiplicities and coupling constants (J_H-H_, Hz), for the *N*-heterocyclic ligands and the corresponding dinuclear platinum(II) complexes **1–3** in D_2_O as a solvent with TSP as the internal standard.

Atom position	^1^H		^13^C	
	qx	**1**	qx	**1**
2,3	8.44, s	9.59, s (+1.15)	145.67	151.77 (+6.10)
5,8	7.51, m	9.73, dd, *J* = 6.7; 3.3 Hz (+2.22)	131.94	135.93 (+3.99)
6,7	7.51, m	8.41, dd, *J* = 6.7; 3.3 Hz (+0.90)	128.83	130.09 (+1.26)
4a,8a	—	—	141.85	145.26 (+3.41)
	qz	**2**	qz	**2**
2	9.09, s	10.12, d, *J* = 11.0 Hz (+1.03)	163.39	178.11 (+14.72)
4	8.84, s	9.52, d, *J* = 8.8 Hz (+0.68)	156.05	168.56 (+12.51)
5	7.80, m	8.51, m	129.07	130.14 (+1.07)
8		(+0.71)	131.53	132.81 (+1.28)
6	7.79, m	8.16, m	130.60	135.11 (+4.51)
7		(+0.37)	138.42	143.10 (+4.68)
4a	—	—	126.98	128.58 (+1.60)
8a	—	—	150.76	152.39 (+1.63)
	phtz	**3**	phtz	**3**
1,4	9.18, s	10.05, s (+0.87)	154.27	164.32 (+10.05)
6,7	7.88, m	8.40, m (+0.52)	136.61	140.50 (+3.89)
5,8	7.88, m	8.40, m (+0.52)	129.38	131.04 (+1.66)
4a,8a	—	—	129.04	130.96 (+1.92)

The aliphatic methylene protons of the bidentatedly coordinated en ligand in **1–3** give a singlet in the region 2.78–2.84 ppm, while the resonance for the corresponding carbon atoms is in the region 49.42–51.27 ppm. s = singlet; d = doublet; dd = doublet of doublets; m = multiplet.

**Table 2:** Comparison of the catalytic activities between **1a** and [{Pt(en)(H_2_O)}_2_(*μ*-pz)]^4+^ [35] and **3a** and [{Pt(en)(H_2_O)}_2_(*μ*-pydz)]^4+^ [44] complexes in the hydrolysis of Ac–L–Met–Gly dipeptide.

Platinum(II) complex	Hydrolyzed Met-Gly amide bond (%)		
	2 h	12 h	24 h
[{Pt(en)(H_2_O)}_2_(*μ*-qx)]^4+^ (**1a**)	35	52	60
[{Pt(en)(H_2_O)}_2_(*μ*-pz)]^4+^	60	85	88
[{Pt(en)(H_2_O)}_2_(*μ*-phtz)]^4+^(**3a**)	8	28	41
[{Pt(en)(H_2_O)}_2_(*μ*-pydz)]^4+^	12	36	54

## Data Availability

Crystallographic data for the structures reported in this manuscript have been deposited with the Cambridge Crystallographic Data Centre under the CCDC number: 1,579,176 (Complex **1**). Copies of these data can be obtained free of charge from http://www.ccdc.cam.ac.uk/data_request/cif.

## Supplementary Materials

Supplementary Materials Figure S1: the UV-Vis spectra for dinuclear platinum(II) complexes **1–3**, which indicated the same bidentate-bridging coordination mode of the corresponding *N*-heterocycle to the Pt(II) ion. Table S1: crystal data and experimental details for [{Pt(en)Cl}_2_(*μ*-qx)]Cl_2_·2H_2_O complex (**1**). Table S2: the selected bond distances and angles for [{Pt(en)Cl}_2_(*μ*-qx)]Cl_2_·2H_2_O complex (**1**). Table S3: Geometrical parameters describing intermolecular hydrogen bonds. Table S4: Geometrical parameters describing stacking interactions.
