# Supplementary material for: Hydrolysis of Methionine- and Histidine-Containing Peptides Promoted by Dinuclear Platinum(II) Complexes with Benzodiazines as Bridging Ligands: Influence of Ligand Structure on the Catalytic Ability of Platinum(II) Complexes
Source: Bioinorg Chem Appl. 2018 May 8;2018:3294948. doi: 10.1155/2018/3294948 (PMC5967607; doi:10.1155/2018/3294948)
Supplement: Supplementary Materials — Figure S1: the UV-Vis spectra for dinuclear platinum(II) complexes 1–3, which indicated the same bidentate-bridging coordination mode of the corresponding N-heterocycle to the Pt(II) ion. Table S1: crystal data and experimental details for [{Pt(en)Cl}2(μ-qx)]Cl2·2H2O complex (1). Table S2: the selected bond distances and angles for [{Pt(en)Cl}2(μ-qx)]Cl2·2H2O complex (1). Table S3: Geometrical parameters describing intermolecular hydrogen bonds. Table S4: Geometrical parameters describing stacking interactions. [file 3294948.f1.doc]

**Hydrolysis of methionine- and histidine-containing peptides promoted by dinuclear platinum(II) complexes with** **benzodiazines as bridging ligands: influence of ligand structure on the catalytic ability of platinum(II) complexes**

Snežana Rajković^1*^, Beata Warżajtis^2^, Marija D. Živković^3^, Biljana Đ. Glišić^1^, Urszula Rychlewska^2^, and Miloš I. Djuran^4^

*^1^University of Kragujevac, Faculty of Science, Department of Chemistry, R. Domanovića 12,* *34000 Kragujevac, Serbia*

*^2^Faculty of Chemistry, Adam Mickiewicz University, Umultowska 89B, 61-614 Poznań, Poland*

*^3^University of Kragujevac, Faculty of Medical Sciences, Department of Pharmacy, Svetozara Markovića 69, 34000 Kragujevac, Serbia*

*^4^Serbian Academy of Sciences and Arts, Knez Mihailova 35, 11000 Belgrade, Serbia*

*Correspondence should be addressed to Snežana Rajković; snezana@kg.ac.rs

**Abstract**

Dinuclear platinum(II) complexes, [{Pt(en)Cl}_2_(*μ*-qx)]Cl_2_^.^2H_2_O **(1)***,* [{Pt(en)Cl}_2_(*μ*-qz)](ClO_4_)_2_ **(2)** and [{Pt(en)Cl}_2_(*μ*-phtz)]Cl_2_^.^4H_2_O **(3)**, were synthesized and characterized by different spectroscopic techniques. The crystal structure of **1** was determined by single-crystal X-ray diffraction analysis, while DFT M06-2X method was applied in order to optimize the structures of **1**−**3**. The chlorido Pt(II) complexes **1**−**3** were converted into the corresponding aqua species **1a**−**3a** and their reactions with an equimolar amount of Ac–L–Met–Gly and Ac–L–His–Gly dipeptides were studied by ^1^H NMR spectroscopy in the pH range 2.0 < pH < 2.5 at 37 ^o^C. It was found that in all investigated reactions with the Ac–L–Met–Gly dipeptide, the cleavage of the Met–Gly amide bond had occurred, but complexes **2a** and **3a** showed lower catalytic activity than **1a**. However, in the reactions with Ac–L–His–Gly dipeptide, the hydrolysis of the amide bond involving the carboxylic group of histidine was observed only with complex **1a**. The observed disparity in the catalytic activity of these complexes is thought to be due to different relative positioning of nitrogen atoms in the bridging qx, qz and phtz ligands and consequent variation in the intramolecular separation of the two platinum(II) metal centers.

TABLE OF CONTENTS

| **Figure S1:** The UV-Vis spectra for the of the investigated platinum(II) complexes **1-3** measured in water (c = 5.10^-5^ mol/L). The inserted chart shows a bathochromic shift of the absorbance peak due to π → π* transitions in quinoxaline after its coordination to Pt(II) ion. | **S3** |
| --- | --- |
| **Table S1:** Crystal data for **1**. | **S4** |
| **Table S2:** Selected bond distances (Å) and valence angles (^o^) of the dinuclear platinum(II) complexes **1-3**. | **S5** |
| **Table S3:** Geometry of the hydrogen bonds in **1.** | **S6** |
| **Table S4:** Geometrical parameters describing π∙∙∙π interaction in **1.** | **S7** |

**
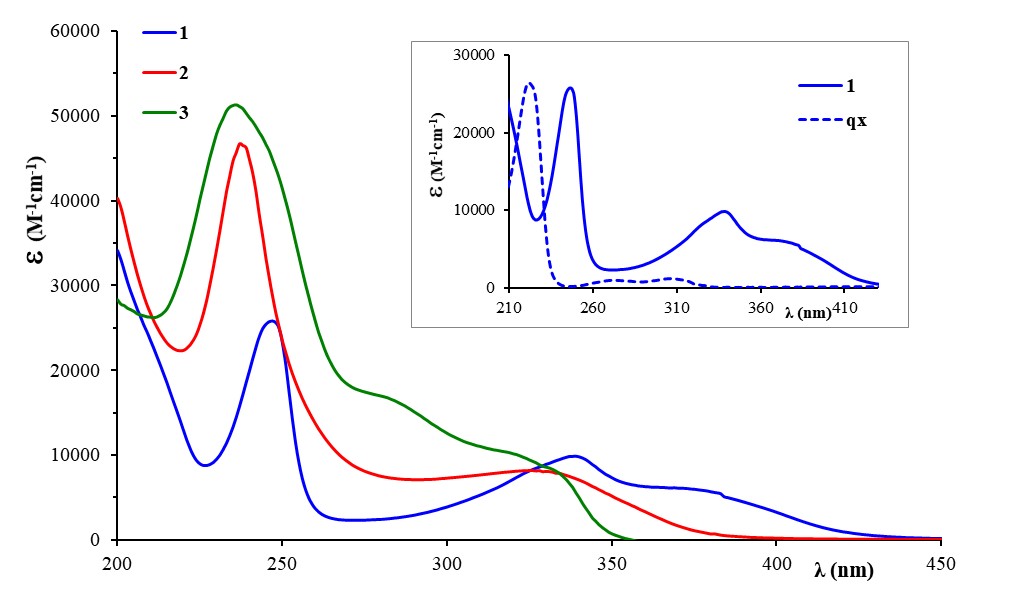
**

**Figure S1:** The UV-Vis spectra for the of the investigated platinum(II) complexes **1-3** measured in water (c = 5.10^-5^ mol/L). The inserted chart shows a bathochromic shift of the absorbance peak due to π → π* transitions in quinoxaline after its coordination to Pt(II) ion.

**Table S1**: Crystal data for **1**.

|  | [{Pt(en)Cl}_2_(*μ*-qx)]Cl_2_**⋅**2H_2_O (**1)** |
| --- | --- |
| Crystal data | |
| Chemical formula | C_12_H_22_Cl_2_N_6_Pt_2_·2(Cl)·2(H_2_O) |
| *M*_r_ | 818.37 |
| Crystal system, space group | Monoclinic, *C*2/*c* |
| Temperature (K) | 295 |
| *a*, *b*, *c* (Å) | 34.190(2), 6.4872(3), 23.087(2) |
| β (°) | 122.744(9) |
| *V* (Å^3^) | 4306.9(6) |
| *Z* | 8 |
| *D_x_* (Mg m^-3^) | 2.524 |
| Radiation type | Mo *K*α |
| μ (mm^-1^) | 13.49 |
| Crystal shape | Prismatic |
| Colour | Yellow |
| Crystal size (mm) | 0.30 × 0.12 × 0.07 |
| Data collection | |
| Absorption correction | Analytical |
| *T*_min_, *T*_max_ | 0.313, 0.600 |
| No. of measured, independent and  observed [*I* > 2σ(*I*)] reflections | 8511, 4659, 3635 |
| *R*_int_ | 0.024 |
| (sin θ/λ)_max_ (Å^-1^) | 0.674 |
| Refinement | |
| *R*[*F*^2^ > 2σ(*F*^2^)], *wR*(*F*^2^), *S* | 0.027, 0.060, 1.03 |
| No. of reflections | 4659 |
| No. of parameters | 235 |
| H-atom treatment | H-atom parameters constrained |
| Δρ_max_, Δρ_min_ (e Å^-3^) | 0.81, -1.18 |

**Table S2**: Selected bond distances (Å) and valence angles (^o^) of the dinuclear platinum(II) complexes **1-3**.

|  | [{Pt(en)Cl}_2_(*μ*-qx)]^2+^  (**1)** | | [{Pt(en)Cl}_2_(*μ*-qz)]^2+^ (**2)** | [{Pt(en)Cl}_2_(*μ*-phtz)]^2+^ (**3)** |
| --- | --- | --- | --- | --- |
|  | X-ray | DFT-calculated | DFT-calculated | DFT-calculated |
| Pt1—N1 | 2.015(4) | 2.0390 | 2.0370 | 2.0348 |
| Pt1—N2 | 2.036(4) | 2.0621 | 2.0621 | 2.0549 |
| Pt1—N5 | 2.024(4) | 2.0519 | 2.0496 | 2.0497 |
| Pt1—Cl1 | 2.2866(14) | 2.3303 | 2.3289 | 2.3480 |
| Pt2—N3 | 2.011(4) | 2.0400 | 2.0394 | 2.0348 |
| Pt2—N4 | 2.034(4) | 2.0652 | 2.0632 | 2.0549 |
| Pt2—N6 | 2.029(4) | 2.0496 | 2.0602 | 2.0497 |
| Pt2—Cl2 | 2.2971(14) | 2.3227 | 2.3296 | 2.3481 |
|  |  |  |  |  |
| N1—Pt1—N2 | 83.25(18) | 83.30 | 83.39 | 83.53 |
| N1—Pt1—N5 | 179.01(17) | 178.29 | 178.51 | 176.90 |
| N2—Pt1—N5 | 95.78(18) | 95.29 | 95.20 | 93.37 |
| N1—Pt1—Cl1 | 90.66(13) | 91.82 | 92.10 | 92.00 |
| N2—Pt1—Cl1 | 173.51(13) | 175.04 | 175.48 | 175.52 |
| N5—Pt1—Cl1 | 90.32(13) | 89.57 | 89.31 | 91.11 |
| N3—Pt2—N4 | 83.62(18) | 83.29 | 83.40 | 83.53 |
| N3—Pt2—N6 | 177.06(17) | 176.83 | 177.77 | 176.90 |
| N4—Pt2—N6 | 93.53(17) | 94.23 | 94.81 | 93.37 |
| N3—Pt2—Cl2 | 91.15(14) | 92.07 | 91.94 | 92.00 |
| N4—Pt2—Cl2 | 174.67(13) | 175.34 | 175.12 | 175.52 |
| N6—Pt2—Cl2 | 91.71(12) | 90.39 | 89.88 | 91.10 |

**Table S3**: Geometry of the hydrogen bonds in **1.**

| *D*—H···*A* | *D*—H (Å) | H···*A* (Å) | *D*···*A* (Å) | *D*—H···*A* (°) |
| --- | --- | --- | --- | --- |
| N1—H1C···Cl4 | 0.89 | 2.28 | 3.165 (5) | 170 |
| N1—H1D···Cl3^i^ | 0.89 | 2.46 | 3.325 (5) | 164 |
| N2—H2C···Cl3^ii^ | 0.89 | 2.37 | 3.209 (5) | 158 |
| N2—H2D···Cl4^iii^ | 0.89 | 2.70 | 3.385 (5) | 135 |
| N3—H3D···Cl3^iii^ | 0.89 | 2.49 | 3.281 (5) | 148 |
| N3—H3C···O1W^iv^ | 0.89 | 2.04 | 2.888 (6) | 159 |
| N4—H4C···Cl3 | 0.89 | 2.90 | 3.548 (5) | 132 |
| N4—H4D···Cl2^v^ | 0.89 | 2.92 | 3.410 (5) | 117 |
| N4—H4D···O2W^vi^ | 0.89 | 2.16 | 2.946 (7) | 147 |
| O1W—H1W···Cl4 | 0.85 | 2.21 | 3.057 (5) | 171 |
| O1W—H2W···Cl3^vii^ | 0.85 | 2.39 | 3.193 (5) | 158 |
| O2W—H3W···O1W | 0.85 | 2.06 | 2.878 (7) | 161 |
| O2W—H4W···Cl4^v^ | 0.85 | 2.44 | 3.152 (5) | 141 |
| C3—H3B···Cl3^viii^ | 0.97 | 2.93 | 3.828 (7) | 155 |
| C4—H4B···Cl3 | 0.97 | 2.82 | 3.436 (6) | 122 |
| C5—H5···Cl2^i^ | 0.93 | 2.71 | 3.432 (5) | 135 |
| C6—H6···Cl1^ii^ | 0.93 | 2.91 | 3.758 (6) | 152 |
| C9—H9···O2W^iii^ | 0.93 | 2.61 | 3.389 (7) | 142 |
| C10—H10···Cl2ix | 0.93 | 2.88 | 3.799 (6) | 170 |

Symmetry code(s): (i) -*x*+1/2, *y*+1/2, -*z*+1/2; (ii) -*x*+1/2, *y*-1/2, -*z*+1/2; (iii) *x*, *y*-1, *z*; (iv) -*x*+1/2, -*y*+1/2, -*z*; (v) *x*, *y*+1, *z*; (vi) -*x*+1/2, -*y*+3/2, -*z*; (vii) *x*+1/2, *y*+1/2, *z*; (viii) -*x*, -*y*, -*z*; (ix) -*x*+1/2, -*y*-1/2, -*z*.

**Table S4**: Geometrical parameters describing π∙∙∙π interaction in **1.**

| h (Å) | C_g_∙∙∙C_g_ (Å) | r (Å) | θ (°) | Symmetry code |
| --- | --- | --- | --- | --- |
| 2.997 | 3.850 | 2.417 | 0.0 | 1/2-x, -1/2-y, -z |
| 3.027 | 3.761 | 2.232 | 0.0 | 1/2-x, 1/2-y, -z |

h = plane∙∙∙plane distance defined as a distance between the center of one ring and a plane defined by the adjacent ring.

Cg = center of gravity (centroid) of the six-membered ring.

Cg∙∙∙Cg = centroid∙∙∙centroid distance.

r = displacement parameter defined as a distance between the centers of the two rings in stack projected on a plane defined by atoms of one of the two rings.

θ = inclination of one ring plane in relation to the other ring plane.
